# Pancreas-enriched miRNAs are altered in the circulation of subjects with diabetes: a pilot cross-sectional study

**DOI:** 10.1038/srep31479

**Published:** 2016-08-25

**Authors:** Attila A. Seyhan, Yury O. Nunez Lopez, Hui Xie, Fanchao Yi, Clayton Mathews, Magdalena Pasarica, Richard E. Pratley

**Affiliations:** 1 Translational Research Institute for Metabolism and Diabetes, Florida Hospital, Orlando, FL, USA; 2 MIT Research Affiliate, Department of Chemical Engineering, Massachusetts Institute of Technology, Cambridge, MA, USA; 3 Sanford Burnham Medical Research Institute, Orlando, FL, USA; 4 Department of Pathology, Immunology, and Laboratory Medicine, University of Florida, Gainesville, FL, USA; 5 College of Medicine Hospital, University of Central Florida, Orlando, FL, USA

## Abstract

The clinical presentation of diabetes sometimes overlaps, contributing to ambiguity in the diagnosis. Thus, circulating pancreatic islet-enriched microRNAs (miRNAs) might be useful biomarkers of β-cell injury/dysfunction that would allow more accurate subtyping of diabetes. We measured plasma levels of selected miRNAs in subjects with prediabetes (n = 12), type 2 diabetes (T2D, n = 31), latent autoimmune diabetes of adults (LADA, n = 6) and type 1 diabetes (T1D, n = 16) and compared them to levels in healthy control subjects (n = 27). The study was conducted at the Translational Research Institute for Metabolism and Diabetes (TRI-MD), Florida Hospital. MiRNAs including miR-375 (linked to β-cell injury), miR-21 (associated with islet inflammation), miR-24.1, miR-30d, miR-34a, miR-126, miR-146, and miR-148a were significantly elevated in subjects with various forms of diabetes compared to healthy controls. Levels of several miRNAs were significantly correlated with glucose responses during oral glucose tolerance testing, HbA_1c_, β-cell function, and insulin resistance in healthy controls, prediabetes, and T2D. These data suggest that miRNAs linked to β-cell injury and islet inflammation might be useful biomarkers to distinguish between subtypes of diabetes. This information could be used to predict progression of the disease, guide selection of optimal therapy and monitor responses to interventions, thus improving outcomes in patients with diabetes.

Diabetes is heterogeneous with respect to genetics, pathophysiology and clinical progression^1^. Regardless of etiology, all forms of diabetes are characterized by either absolute or relative defects in insulin secretion. At one end of the spectrum, T1D is characterized by autoimmune destruction of β-cells resulting in a total or near-total loss of β-cell mass and insulin secretory capacity. Even within this group there is heterogeneity, however, those with evidence of residual insulin secretion manifest better glycemic control and improved outcomes. At the other end of the spectrum, patients with T2D whose β-cell mass is ~40% of normal on average continue to secrete significant, albeit inadequate, amounts of insulin. In between these extremes, LADA onset has genetic and clinical features typical of both T1D and T2D. Because of overlap in the clinical presentation of these syndromes, individuals are sometimes misdiagnosed, resulting in delayed initiation of appropriate therapy. For example, it is not uncommon for patients with LADA to go several months before their requirement for insulin is recognized. Increases in obesity in the general population, coupled with a rise in the incidence of T2D in youth, have also made it increasingly difficult to subtype diabetes on purely clinical grounds.

A major gap in the field of diabetes is that we have not identified appropriate biomarkers^2^,^3^ that relate to the underlying pathophysiology of β-cell destruction and β-cell mass. A variety of measures of insulin secretion including fasting indices, oral and intravenous glucose tolerance tests and other provocative challenges are useful to gauge β-cell function. These tests have been used to document defects in insulin secretion and predict progression in subjects before the onset of both T1D and T2D. Although measures of β-cell function are commonly performed in research studies, they have not achieved widespread clinical use, in part because testing is time consuming and expensive and the assays are not standardized. These measures are also poorly correlated to β-cell mass in general and do not provide insight into the pathophysiology underlying β-cell dysfunction. To gauge autoimmune-mediated β-cell injury, islet autoantibodies (aAbs) and measurement of T-cell reactivity are useful and are often detectable before T1D develops^4^. However, they do not predict disease onset and cannot be used to monitor disease progression. While a number of groups are exploring imaging methods for monitoring β-cell mass, morphometric analyses of autopsy specimens is currently the only way to measure β-cell mass in humans. Therefore, better biomarkers of β-cell injury and mass are needed to gain insights into disease pathophysiology, assess disease activity, personalize therapy and monitor responses to treatment.

Altered levels of circulating miRNAs have been associated with a variety of conditions (*e*.*g*., cancer, metabolic and cardiovascular diseases, neurodegenerative diseases, autoimmunity, and aging)^2^. The miRNA species in the circulation may reflect the activation state of circulating cells or tissue injury in response to disease states. For example, decreased levels of miR-126, miR-15a, and miR-223 are detectable several years before the onset of T2D^5^; whereas, miR-9, miR-29a, miR-30d, miR-34a, miR-124a, miR-146a, and miR-375 were shown to be increased in newly diagnosed T2D patients compared with persons with prediabetes^6^.

We hypothesized that circulating islet-specific miRNAs would be useful biomarkers of β-cell injury and that this might be useful to subtype diabetes. To test this, we compared plasma expression levels of a panel of 28 miRNAs (Supplementary Table 1) associated with islet function or previously associated with diabetes^2^ in subjects with prediabetes, T2D, LADA, and T1D to those of healthy controls.

## Results

### Clinical and metabolic characteristics of the study population

The demographic, clinical and metabolic characteristics of the individual groups of subjects are detailed in Table 1. Due to the imbalances in BMI, age, and gender, all further analyses corrected for these confounding variables. HbA_1c_ was higher in patients with diabetes. Diabetes was well controlled in subjects with T2D and T1D (mean HbA_1c_ levels of 6.6% and 7.6%, respectively) but was significantly worse among those with LADA (HbA_1c_ levels of 8.8%). Mean HbA_1c_, baseline glucose, and baseline insulin levels for the prediabetes group were not significantly different from the mean healthy control levels because the prediabetes group included a heterogeneous subset of individuals at increased risk for diabetes (*i*.*e*., subjects with IFG, or IGT, or with just a mild increase in HbA_1c_ level). Because these subjects have altered values in just one or the other measured magnitudes, the net effect in the respective averaged value for the group is reduced to statistically non-significantly different values. However, we were able to capture significant differences in the insulin secretion-sensitivity index-2 (ISSI2) for this group as compared to the healthy control group. ISSI2 has been used as an OGTT-based measure of β-cell function. Ideally, subsets of subjects with IFG, IGT, and mildly altered HbA_1c_ should be analyzed by separate, but this could not be done in our study due to the limited sample size of the respective subgroups. Consistent with the HbA_1c_ levels, the glucose area under the curve (AUC) during the OGTT increased progressively from healthy controls to prediabetes to T2D/T1D and was highest in those with LADA. Fasting C-peptide concentrations, indicative of basal insulin secretion, were highest in subjects with prediabetes and T2D and lowest in those with LADA and T1D as expected. Indices of insulin secretion and action from the OGTT were only calculated in healthy controls, prediabetes and T2D subjects. As expected, the groups with prediabetes and T2D were significantly more insulin resistant, although HOMA-B and the insulinogenic index did not differ between these groups.

### Pancreatic islet-enriched miRNAs are elevated in the circulation of subjects with diabetes

**Figure 1:**
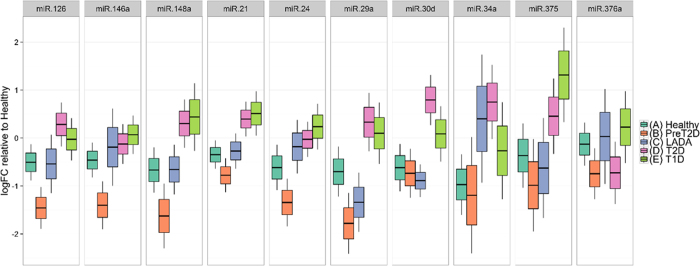
Circulating levels of pancreas-enriched miRNAs in subjects with different types of diabetes. The prediabetes group showed significant reduced levels of miR-126 and miR-146a (*p* < 0.05, FDR < 0.15). The group with type 2 diabetes exhibited significantly elevated levels of circulating miR-30d, miR-34a, miR-21, and miR148a (*p* < 0.05, FDR < 0.1). The group with T1D exhibited significantly higher levels of miR-21, miR-375 (associated with β-cell death), miR-148a, and miR-24.1 (associated with islet-inflammation) (*p* < 0.05, FDR < 0.05). The fold change for every miRNA was obtained using the 2^−∆∆Ct^ method^24^,^25^ with the geometric mean^26^ of two endogenous control miRNAs (hsa-miR-191 and miR-451) for normalization. The data are presented as log fold-change ± SEM (box) and the 95% confidence interval (whiskers). The limma R package was used to assess the statistical significance of miRNA differential abundance in circulation. Data was adjusted for confounding variables: BMI, age, and gender. Statistical significance was considered at *p*-value < 0.05. Corresponding *p* and adjusted *p*-values are reported in Table 2.

Of the 28 miRNAs initially selected for profiling, eight pancreas-enriched miRNAs showed significantly altered levels in the plasma of subjects with various forms of diabetes as compared to healthy controls (p < 0.05, FDR < 0.15, Table 2, Fig. 1, Supplementary Table 2). The pattern of expression differed markedly among miRNAs. Both, miR-21 and miR-148a, for example, were significantly increased in T2D and T1D relative to healthy controls, but levels of these two miRNAs were not significantly different among these subtypes of diabetes. In contrast, miR-24 and miR-375 were significantly elevated in T1D only, while miR-30d and miR-34a were only significantly elevated in T2D, as compared to healthy controls (Table 2, Fig. 1). On the other hand, levels of miR-126 and miR-146a were reduced in subjects with prediabetes and no significant differential abundance was detected in the LADA group as compared to healthy controls (Fig. 1). Interestingly, the subjects with prediabetes showed a general trend to reduction of the circulating miRNA levels, while subjects at later stages of the disease (*e*.*g*., T2D and T1D) showed a trend to increased levels of the circulating pancreatic miRNAs.

We tested the reproducibility of detection and effects of acute increase in glucose on the stability of circulating miRNAs. The miRNAs were stable in fasting plasma samples collected on two different days (CV: 4%) and they did not change with acute changes in glucose during the OGTT (CV: 5%) in T2D subjects (Supplementary Fig. 1).

### Unique signatures of circulating miRNAs are associated with different subtypes of diabetes

Differential abundance analysis (Table 2, Fig. 1) and Random Forest (RF) classification was used to determine whether control subjects could be distinguished from cohorts with different types of diabetes based on their miRNA profile. In this case, miRNA expression profiles were compared to the clinical classification of the subjects. Such models could enable the creation of panels of miRNAs with the highest discriminatory capacity for each class, thus making it possible to identify the miRNA signatures that best differentiate between classes. Differential abundance analysis revealed that different subtypes of diabetes have distinct averaged miRNA signature profiles (Table 2). For example, miR-375 and miR-24 were significantly different only in T1D, while miR-30d and miR-34a, were only significantly different in T2D, as compared to healthy controls. On the other hand, miR-146a and miR-126 were significantly downregulated in people with prediabetes. Two miRNAs (*i*.*e*., miR-21 and miR-148a), were similarly significantly elevated in people with diabetes, either T2D or T1D. These data suggest that pancreatic miRNAs elevated in circulation (*e*.*g*., miR-375, miR-24, miR-21, and miR-148a) may better represent the degree and severity of β-cell injury that is found greater in T1D than T2D, than LADA and prediabetes. Due to the limited number of subjects with LADA in our study, we were unable to detect statistically significant differences between this subgroup and the healthy control group. However, we observed a trend for miR-34a to be elevated as in subjects with T2D.

**Figure 2:**
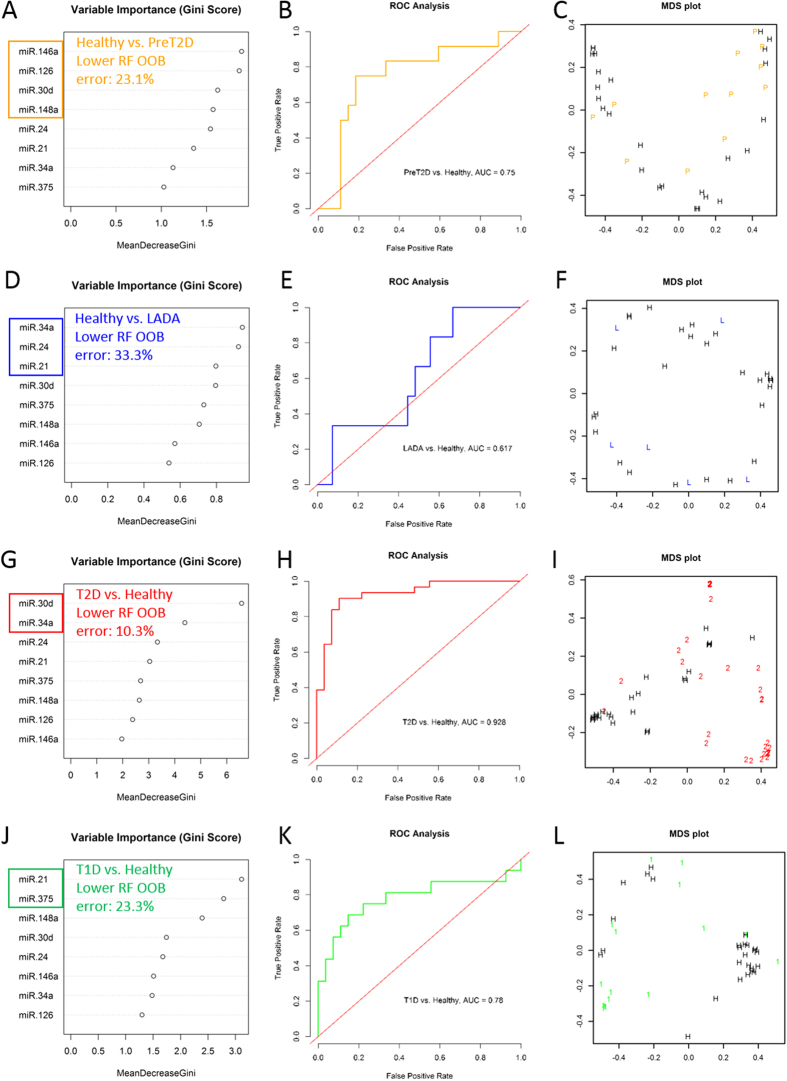
Binary RF classification of diabetes subtypes *versus* Healthy designation. Random Forest (RF) classification was performed in the R environment using the randomForest package. Data used was BMI, age, and gender-adjusted logFC for differentially abundant circulating miRNAs and the clinical designation (class label) of each individual [*i*.*e*., Healthy, Prediabetes, LADA, T2D, and T1D]. Four sets of binary classifications were conducted, one for each diabetes subtype as compared to Healthy control group. (**A**–**C**) shows the results for Prediabetes *vs*. Healthy classification. (**D**–**F**) shows the results for LADA *vs*. Healthy classification. (**G**–**I**) shows the results for T2D *vs*. Healthy classification. (**J**–**L**) shows the results for the T1D *vs*. Healthy classification. Left panels display the variable importance plot (Gini scores) determined during the initial binary RF classification including all 8 differentially abundant circulating miRNAs. This order of variable importance was used to recursively repeat the RF classification including the top 2, 3, and so forth combinations of miRNAs as predictor variables, and identify the binary classifier with the lower out-of-bag (OOB) estimate of error rate. Outline-colored boxes enclose the combination of miRNAs that generated the classifier with the lower OOB error rate (reported in the top left corner of each left panel graph). The middle panels display the Receiver Operator Characteristic (ROC) Curve generated for sensitivity analysis using the ROCR package. The RF prediction probabilities were used for the generation of the ROCR prediction object. The area under the curve (AUC) is reported as performance measure. The right panels display the multidimensional scaling (MDS) plots for each respective binary RF classification. Color and symbol coding: black and H: Healthy group; orange, P, and PreT2D: Prediabetes group; blue and L: LADA group; red and 2: T2D group; green and 1: T1D group.

To validate the existence of such miRNA signatures based on differential abundance in circulation, we implemented an RF binary classification approach to identify the miRNA combinations that best separate each disease group from the Healthy group. RF generates importance measures for the features used as predictor variables (miRNA levels in our case), which are helpful for feature selection. Based on the Gini scores of variable importance extracted from an initial RF run including eight differentially abundant miRNAs (Fig. 2, left panels), we recursively generated distinct RF classifiers with distinct miRNA combinations and evaluated their performances to identify those with the lower out-of-bag (OOB) estimates of error rates for each binary classification. This approach identified four distinct combinations of miRNAs that can distinguished each disease subtype from healthy controls, with relatively low OOB estimates of error rate and with relatively high area under the curve (AUC) in sensitivity analysis (except for the LADA group, Fig. 2 middle panels). In specific: (1) subjects with prediabetes were best distinguished from healthy controls using a binary RF classifier based on the circulating levels of 4 miRNAs: miR-146a, miR-126, miR-30d, and miR-148a (OOB estimate of error rate = 23.1%); (2) people with LADA were best distinguished based on the levels of miR-34a, miR-24, and miR-21 (OOB estimate of error rate = 33.3%); (3) T2D subjects were best distinguished by the binary classifier evaluating the levels of two circulating miRNAs: miR-30d and miR-34a (OOB estimate of error rate = 10.3%); and (4) people with T1D were best classified based on the circulating levels of miR-21 and miR-375 (OOB estimate of error rate = 23.3%). Multidimensional scaling (MDS) plots from each binary RF classification, presented in the right panels in Fig. 2, demonstrate the separation of subjects into two major classes with certain level of heterogeneity (worst in the LADA *vs*. Healthy classification). Confusion matrices and additional performance measures for each binary classifier are provided in Supplementary Tables 3–8.

### Use of miRNA signatures alone is not robust enough for accurate multi-class classification of diabetes subtypes

**Figure 3:**
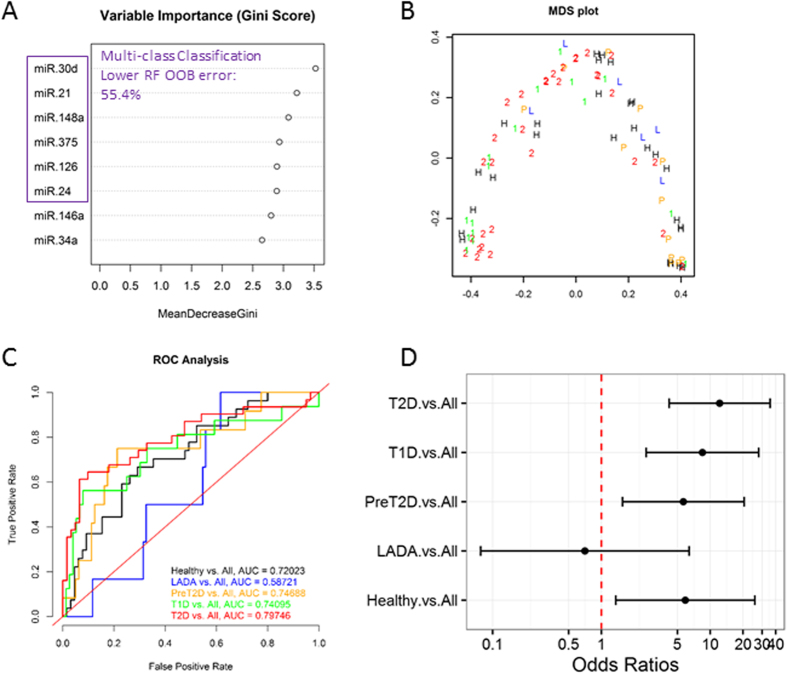
Multi-class RF classification of diabetes subtypes. Random Forest (RF) classification was performed in the R environment using the randomForest package. Data used was BMI, age, and gender-adjusted logFC for differentially abundant circulating miRNAs and the clinical designation (class label) of each individual [*i*.*e*., Healthy, Prediabetes, LADA, T2D, and T1D]. (**A**) Variable importance plot (Gini scores) determined during the initial multi-class RF classification including all 8 differentially abundant circulating miRNAs. This order of variable importance was used to recursively repeat the RF classification including the top 2, 3, and so forth combinations of miRNAs as predictor variables, and identify the binary classifier with the lower out-of-bag (OOB) estimate of error rate. Outline-colored box encloses the combination of miRNAs that generated the classifier with the lower OOB error rate (reported in the top left corner). (**B**) Multidimensional scaling (MDS) plot generated for the multi-class RF classifier that included the top 6 miRNAs and performed with the lower OOB estimate of error rate. (**C**) Receiver Operator Characteristic (ROC) Curves generated by plotting the classifier true positive rate (sensitivity) as a function of the false positive rate (1-specificity). The multi-class RF prediction probabilities were used for the generation of ROCR prediction objects. The 5-class classification problem was reformulated as five one-*versus*-all (OVA) binary comparisons. The area under the curve (AUC) is reported as performance measure. (**D**) Diagnostic Odds Ratios calculated for each OVA comparison using the formula DOR = (TP/FN)/(FP/TN), where TP is the number of true positives, FN, the number of false negatives, FP, the number of false positives, and TN, the number of true negatives. DOR of a test is a single indicator of diagnostic performance and represents the odds of positivity in individuals with the disease relative to the odds of positivity in individuals without the disease. Color and symbol coding: black and H: Healthy group; orange, P, and PreT2D: Prediabetes group; blue and L: LADA group; red and 2: T2D group; green and 1: T1D group.

**Figure 4:**
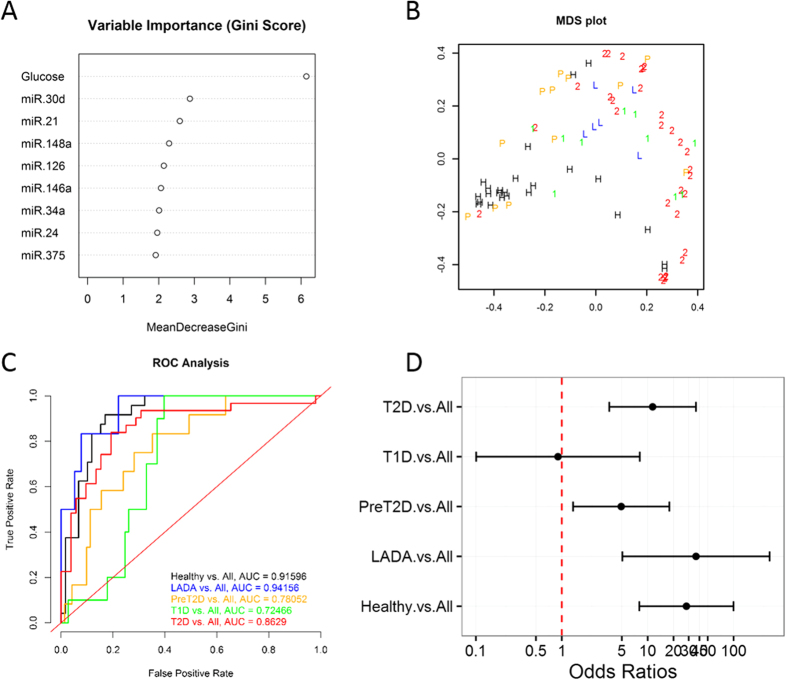
Multimodal multi-class RF classification of diabetes subtypes. Description of this figure is the same as for Fig. 3, with the only difference that the baseline glucose level was included as a predictor variable in addition to circulating miRNA levels.

To assess the real diagnostic value of circulating pancreatic miRNAs, we implemented an alternative RF classification approach to “simultaneously” discriminate among all five study groups (multi-class classification approach). Based on the Gini scores of variable importance extracted from an initial RF run including eight differentially abundant miRNAs (Fig. 3A), we generated distinct multi-class RF classifiers with distinct miRNA combinations. The classifier generated by evaluating a combination of the top six most important miRNAs (*i*.*e*., miR-30d, miR-21, miR-148a, miR-375, miR-24a, and miR-126) was identified as the best performing one, based OOB estimation of error rate. However, at 56.5%, this error rate is prohibitively high for useful clinical subtype differentiation following a multi-class classification approach. Assessment of additional performance measures for the multi-class RF classifier, including ROC AUC, PPV, NPV, and others (see confusion matrices and performance statistics in Supplementary Table 8) demonstrate its limited discriminative power among diabetes subtypes. Although ROC sensitivity analysis consistently produced AUCs in the range 0.59–j6 0.80 (Fig. 3B) and diagnostic odds ratios (DORs) greater than 5 with confident intervals that do not include 1 for all but one classification (Fig. 3D), our results suggest that larger AUCs and DORs are required for accurate classification. In an effort to improve the performance of the multi-class classifier, we included the fasting glucose level (which is a generally available measure taken during routine screening of individuals at risk of diabetes) as an additional predictor variable and demonstrated an improvement in the OOB estimate of error rate (down to 43.4%) and complementary performance measures of the “multimodal” (circulating miRNAs + fasting glucose) multi-class RF classifier (Fig. 4 and Supplementary Table 8).

### Partial correlation analysis underscores significant association of circulating miRNAs with clinically-relevant glucose and insulin parameters/indices

**Figure 5:**
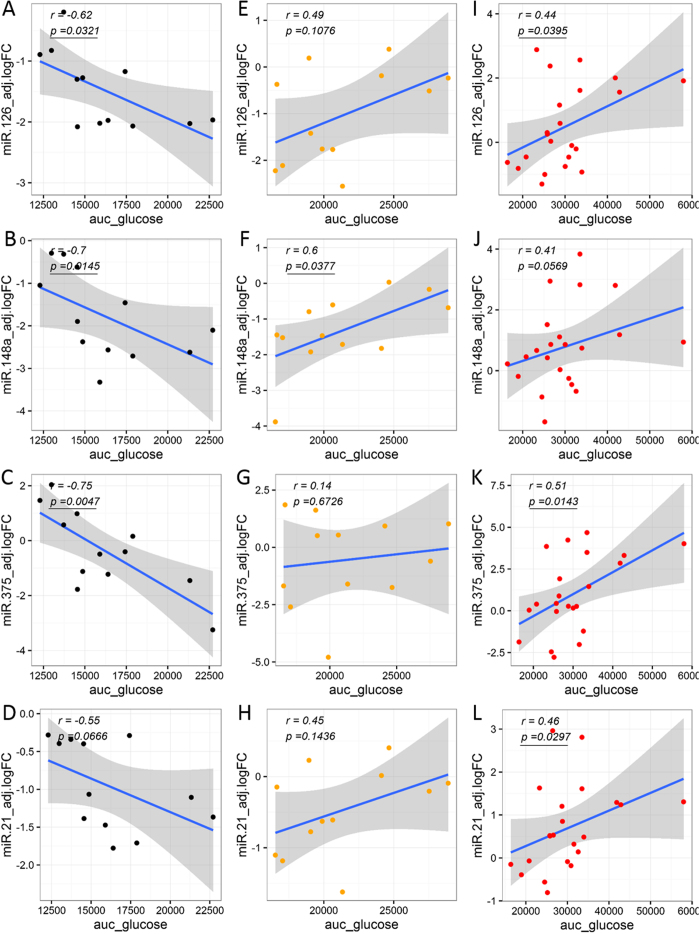
Differential associations between circulating miRNA levels and clinical measure of glycemic control (glucose AUC). Correlation analyses were performed by calculating partial correlation coefficients between clinical parameters and circulating miRNA logFC levels adjusted for BMI, age, and gender. Left panels (A–D) show correlation plots for the Healthy group (black dots). Middle panels (E–H) show correlation plots for the Prediabetes group (orange dots). Right panels (I–L) show correlation plots for the T2D group (red dots). Blue line represent the linear fit of the plotted points, shown for visualization purposes (gray band represent the associated 95% confidence interval of the fit). Partial correlation coefficient (r) reported in top left corner of each plot. Statistical significance was considered at p-value < 0.05 (bold and underline text in left top corner of plot).

**Figure 6:**
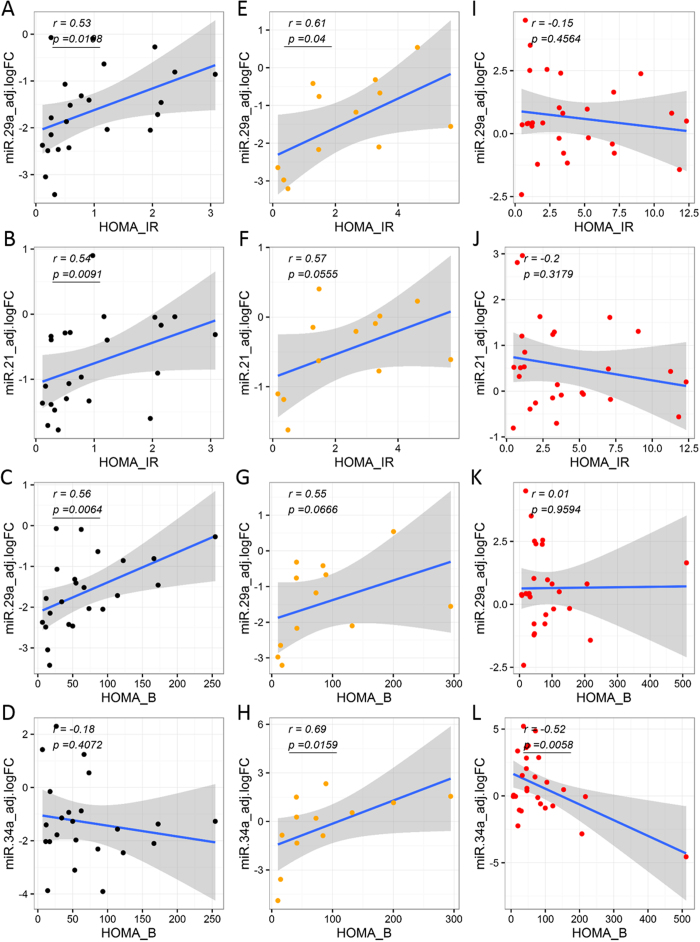
Differential associations between circulating miRNA levels and clinical indices of insulin sensitivity and β-cell function (HOMA IR, HOMA B). Correlation analyses were performed by calculating partial correlation coefficients between clinical parameters and circulating miRNA logFC levels adjusted for BMI, age, and gender. Left panels (A–D) show correlation plots for the Healthy group (black dots). Middle panels (E–H) show correlation plots for the Prediabetes group (orange dots). Right panels (I–L) show correlation plots for the T2D group (red dots). Blue line represent the linear fit of the plotted points, shown for visualization purposes (gray band represent the associated 95% confidence interval of the fit). Partial correlation coefficient (r) reported in top left corner of each plot. Statistical significance was considered at p-value < 0.05 (bold and underline text in left top corner of plot).

**Figure 7:**
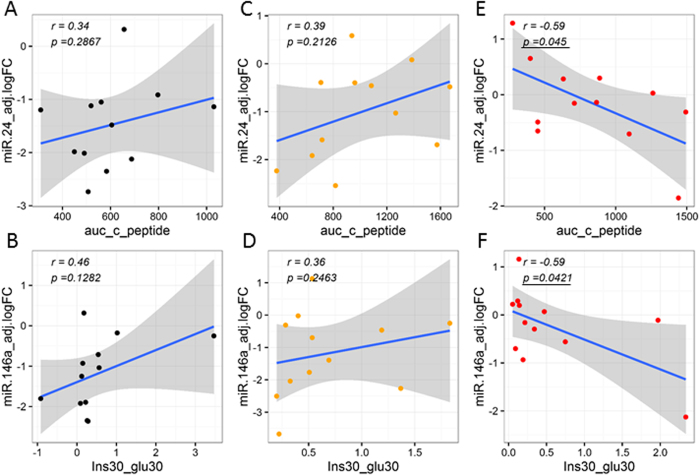
Differential associations between circulating miRNA levels and clinical indices of β-cell function (Insulinogenic index and c-peptide AUC). Correlation analyses were performed by calculating partial correlation coefficients between clinical parameters and circulating miRNA logFC levels adjusted for BMI, age, and gender. Left panels (A–D) show correlation plots for the Healthy group (black dots). Middle panels (E–H) show correlation plots for the Prediabetes group (orange dots). Right panels (I–L) show correlation plots for the T2D group (red dots). Blue line represent the linear fit of the plotted points, shown for visualization purposes (gray band represent the associated 95% confidence interval of the fit). Partial correlation coefficient (r) reported in top left corner of each plot. Statistical significance was considered at p-value p < 0.05 (bold and underline text in left top corner of plot).

The correlation of circulating miRNA abundance levels to measures of glycemic control, insulin secretion, and insulin action was examined for each individual group in the subset of subjects not on exogenous insulin (*i*.*e*., healthy controls, prediabetes, and T2D). In all groups, differentially abundant circulating miRNA levels were significantly correlated with either glycemic control parameters (*i*.*e*., AUC-Glucose and HbA_1c_) and/or β-cell function and/or insulin action indices (i.e., AUC-Insulin, AUC-C-peptide, HOMA-B, HOMA-IR, MATSUDA, QUICKI, ISSI2). Interestingly, the number of significant correlations was higher in the prediabetes group (35 significant correlations, Supplementary Table 9), as compared to the Healthy control and T2D groups (25 and 10 significant correlations, respectively, Supplementary Tables 10 and 11). Notably, we uncovered a switch in the sign of several correlations as we compared the correlations identified in the Healthy group, with those in the prediabetes and T2D groups (Figs 5–7).

## Discussion

The results of this study, correcting for three major confounding variables (*i*.*e*., BMI, age, and gender), demonstrate that abundance levels of eight pancreas-enriched miRNAs (*i*.*e*., miR-375, miR-21, miR-24.1, miR-30d, miR-34a, miR-126, miR-146, and miR-148a) are significantly (*p* < 0.05, FDR < 0.15) altered in the circulation of persons with different types of diabetes, as compared to healthy controls (Table 2, Fig. 1). Notably, the abundance level patterns of circulating miRNAs differed among subtypes of diabetes. For example, miR-375 was only significantly elevated in the plasma of subjects with autoimmune-mediated T1D, while miR-30d was only significantly elevated in T2D. This was confirmed with a binary RF classification approach (Fig. 2), which identified combinations of circulating miRNAs that can be used to separate healthy controls from subjects with a specific diabetes subtype.

Whether the increase in miR-375 in T1D reflects the autoimmune process, *per se*, or ongoing injury to residual β-cells is not completely understood. While individuals with LADA and T1D had the lowest insulin secretory capacity as evidenced by fasting c-peptide levels, as expected, many manifested low, but significant, c-peptide responses to the OGTT, indicative of residual β-cell function. We (data not shown) and others have shown that miR-375 is abundantly expressed in pancreatic islets and involved in β-cell proliferation and glucose dependent insulin and glucagon secretion from β and α-cells, respectively^7^. Increased plasma levels of miR-375 have been linked to β-cell death^8^ and were shown to predict hyperglycemia in mouse models of T1D and in humans with T2D^9^. miR-375 is required for normal glucose homeostasis and its loss in a genetic knockout model resulted in hyperglycemia, increased α-cell mass^7^ and loss of β-cell mass. This miRNA functions in cooperation and/or redundant fashion with other miRNAs^10^. In β-cell cultures, miR-375 inhibits insulin secretion, in part by inhibiting the translation of the Myotrophin^11^ and Pyruvate Dehydrogenase Kinase Isoform 1^12^. Collectively, the data suggest that elevated levels of miR-375 in circulation reflect β-cell injury. However, a recent report indicated that circulating miR-375 levels were also increased in subjects with autoimmune-mediated Hashimoto’s thyroiditis^13^. This might suggest a more general role for miR-375 in autoimmunity, as well as a possible mechanistic link for the well-documented association of T1D and Hashimoto’s thyroiditis. The significant differences among the groups in our study suggest that circulating levels of miR-375 might be a useful biomarker, in addition to autoantibodies, to distinguish individuals with T2D from those with T1D or LADA. In contrast to miR-375, miR-30d and miR-34a were most significantly increased in the group with T2D relative to healthy controls. A recent report found that prolonged exposure of the β-cell line MIN6 to high glucose altered the expression of a number of miRNAs including miR-30d^14^. Overexpression of miR-30d reduced insulin gene expression, suggesting a possible role of this miRNA in defective insulin biosynthesis under diabetic conditions^14^.

In contrast to what occurred in the subgroups representing later stages of the disease, the differentially abundant circulating miRNAs appeared to decrease in the prediabetes stage (*i*.*e*., miR-126 and miR-146a). Some miRNAs also decreased, although not significantly, in the LADA group (Table 2), which somehow could be understood as the “pre-T1D” stage. This result is interesting and possibly hints at adaptive responses taking place in these subjects. An adaptive miRNA-regulated response would be consistent, for example, with an increase in β-cell mass to compensate for the insulin resistance developing in these subjects. The partial correlation data (Supplementary Tables 9–11) additionally support a case for the differentially abundant circulating miRNAs in association with measures of β-cell function. Some of these miRNAs are known to be involved in β-cell growth and apoptosis, insulin secretion, insulin synthesis and endothelial function. For example, miR-34a and miR-146a are elevated in pancreatic islets from diabetic obese mice and significantly affect the survival of β-cells and insulin exocytosis^15^. *In vitro* treatment of an insulin secreting mouse cell line (MIN6B1 cells) and pancreatic islets with palmitate induced miR-34a and miR-146 expression in a dose-dependent manner. Activation of p53 upregulated miR-34a possibly mediating β-cell apoptosis and impairing nutrient-induced insulin secretion. miR-148a is involved in insulin synthesis and blocks insulin expression^15^,^16^. In addition, miR-146a plays an important role in the adaptive immune response by regulating expression of IL-2^17^. Whether the elevation of these miRNAs was secondary to hyperglycemia or reflected other metabolic derangements in the diabetic subjects is not clear. Several of these miRNAs were correlated with glycemic control and HbA_1c_. These miRNAs were also correlated with indices of insulin secretion, insulin resistance, and β-cell function in general (AUC-Insulin, AUC-C-peptide, HOMA-B, HOMA-IR, MATSUDA, QUICKI, ISSI2) in the subset of subjects who were not treated with insulin.

Biomarker potential of this miRNA panel to distinguish among all subtypes of diabetes included in our study was further assessed using a multi-class RF classification approach. The RF algorithm assigns importance scores to each miRNA depending on how well they perform during the classification. The best performing multi-class RF classifier evaluated the six most important differentially abundant circulating miRNAs (*i*.*e*., miR-30d, miR-21, miR-148a, miR-375, miR-24, and miR-126) and yielded receiver operating characteristic (ROC) curves with AUC values ranging from 0.59 to approximately 0.80 and DOR ratios greater than 5 and not including 1 in the 95% confident interval for all classifications but for LADA *vs*. all other subtypes (Fig. 3 and Supplementary Table 6). Although the overall performance of the multi-class classifier indicated that the differentially abundant circulating miRNAs have potential as biomarkers, the use of miRNA signatures alone was not robust enough for accurate diagnosis of diabetes subtypes. Indeed, we demonstrated that by including fasting glucose levels as an additional predictor in the multimodal multi-class RF classifier, we can improve the OOB error rate and performance measures (Fig. 4, Supplementary Table 8). Therefore, we reason that RF classifiers including a variety of predictor variable types (*e*.*g*., circulating miRNA levels, fasting glucose, fasting insulin or c-peptide measures, and presence of autoantibodies, among others) could reach an optimal performance for accurate diagnosis of diabetes subtypes. A multimodal biomarker approach refers to the use of a combination of two or more biomarker modalities in a verification/identification system. The advantage with this approach is that biomarkers representing different underlying pathophysiology and mechanisms often lead to better diagnosis and prognosis. However, the approach is susceptible to noise. This can lead to inaccurate matching, as noisy data may lead to a false rejection. Conversely, unimodal biomarkers refers to the use only one biomarker for verification/identification and often represents a single pathophysiologic mechanism. In this case, the biomarker’s traits might be noisy or distorted leading to false or non-specific positives. Although our classifiers performed sub-optimally, probably influenced by the limited sample sizes in our study and the known large phenotypic heterogeneity of the diabetes subtypes, these results warrant additional efforts in larger cohort and longitudinal studies to better assess the clinical utility of a diabetes biomarker RF classifier. The nonsignificant DOR for the classification of LADA *vs*. all other groups (Fig. 3D) and the lack of significant differential miRNA abundance in the circulation of subjects with LADA (Table 2) reflects the limited power of our study due to the reduced sample size of the LADA group. However, as shown in Fig. 1 and the binary RF classification approach (Fig. 2B), miRNAs like miR-34a, miR-30d, and miR-24 could be useful to classify subjects with LADA, which are usually misdiagnosed as T2D but quickly advance to T1D stage. By better understanding the disease evolution in these subgroups, we will be able to better manage delaying or even halting development of advanced disease.

Our study provides valuable insights into the molecular characterization of prediabetes and to a lower extent (due to limited sample size) into the characterization of LADA. Both subtypes represents a transitional state, with metabolic abnormalities typical of T2D in the prediabetes state, and typical of T1D in the LADA state. The diagnosis of prediabetes can be made on the basis of the fasting plasma glucose, the 2-hour glucose during an OGTT or the HbA_1c_, while for LADA, autoantibodies need to be additionally detected. However, it would be extremely valuable to identify these subjects before clinical symptoms appears, in order to better manage strategies that delay β-cell destruction. miRNAs have been suggested as good candidates for early diagnosis of diseases and some already detected several years before T2D development (*i*.*e*., miR-126 in prediabetes)^5^,^18^. In this study, we found that several miRNAs that were elevated in the plasma of diabetic subjects were also found changing in the plasma of prediabetes and LADA subjects, but interestingly, in the opposite direction (Table 2, Fig. 1) [*e*.*g*., miR-126 and miR-146a levels were reduced in prediabetes (*p* < 0.05), and non-significantly miR-29a, miR-375, and miR-30d in LADA]. We speculate that these changes may reflect adaptive responses during early stages of diabetes development and therefore could have potential for early diagnosis. This also suggests that the increased abundance in circulation in later stages of disease development underlies the pathophysiology of insulin resistance and β-cell dysfunction.

Notably, our correlation analysis among circulating miRNA levels and relevant clinical measures/indices of glycemic control and β-cell function, we uncovered a switch in the sign of several correlations as we compared relevant partial correlations calculated for each independent group not subjected to exogenous insulin therapy (Healthy, Prediabetes, and T2D groups). One of the most striking changes, in our opinion, was the switch from negative to positive correlation between circulating miRNA levels and glucose AUC calculated from the OGTT (Fig. 5). In the Healthy group, as the circulating miRNA levels increase, the glucose AUC decreases, indicating an association between an enhanced ability for glucose disposal (glucose tolerance) in healthy subjects with increased levels of miR-126, miR-148a, miR-29a, and miR-375 (Fig. 5A–D, Supplementary Table 10). On the contrary, in subjects with prediabetes, elevated miRNA levels (*i*.*e*., significantly for miR-148a, Fig. 5F, Supplementary Table 9) correlated with elevated measures of glucose AUC (Fig. 5E–H). Similarly, elevated levels of miR-126, miR-21, miR-30d, and miR-375, significantly and positively correlated with glucose AUC in the T2D group (Fig. 5I–L, Supplementary Table 11). These results suggest the development of glucose intolerance in association with increases in the levels of specific circulating miRNAs in people with prediabetes and T2D. On the other hand, elevated levels of specific circulating miRNAs (including some of those mentioned above) significantly associated with increased levels of insulin resistance (*i*.*e*., higher HOMA-IR and lower QUICKI) and insulin secretion (*i*.*e*., higher HOMA-B) in people from the Healthy and Prediabetes groups (Fig. 6A–C,E,H, Supplementary Tables 9 and 10). This suggests that the elevation of pancreas-enriched miRNAs in the circulation of healthy subjects and subjects with prediabetes is associated with an augmented activity of pancreatic β-cells as the healthy individual (or the individual in an early stage of disease development) tries to compensate for reduced insulin sensitivity. Importantly, the contrary occurred in the T2D group (Supplementary Table 11), where higher levels of circulating miR-24, miR-34a, and miR-146a negatively correlated with c-peptide AUC (Fig. 7E), HOMA-B (indicator of β-cell activity/insulin secretion, Fig. 6L), and the insulinogenic index (ΔIns30/ΔGlu30, indicator of early-phase insulin secretion in response to glucose, Fig. 7F), respectively. This suggests that the elevation of pancreas-enriched miRNA levels in the circulation of people with T2D is not associated with an enlarged capacity to produce and secrete insulin as in healthy subjects and people with prediabetes. Rather, the increase in circulating levels of these miRNAs in people with T2D is likely due to increased β-cell death accompanied by release of intracellular contents. We want to note that the positive correlation detected between elevated levels of miR-146a and miR-24 with increased values of the MATSUDA index (apparently indicating improvement of insulin sensitivity with increased miRNA levels) in people with T2D is likely due to an artifact in the MATSUDA calculation. This could be ascribed to the reduction of the insulin response to glucose due to β-cell dysfunction.

Overall, our results suggest that different types of diabetes have unique molecular signatures that could be useful (although not sufficient on their own) for subtyping diabetes. The rich information content of miRNAs, their relative tissue specificity and their stability^19^ in biological samples suggests that they might be good, minimally invasive, and cost-effective biomarkers of β-cell dysfunction in diabetes. Nevertheless, a number of scientific and technical considerations must be addressed. First, since cohorts analyzed in the present study were small and the analyses cross-sectional in nature, validation studies with larger numbers of subjects and longitudinal follow-up are warranted. Second, despite concerns of long-term stability of miRNAs in archival samples, all evidence suggest that miRNAs are highly stable in blood and other bodily fluids for multiple freeze-thaw cycles^19^,^20^ and over as many as 5 years at −20 °C^20^. We have successfully measured miRNAs and inflammatory cytokines in banked sample that were >5 years old and were able to differentiate between T2D groups under different drug treatments (unpublished data). Third, since changes in the levels of circulating miRNAs as biomarkers do not necessarily reflect dysregulation of miRNA expression within β-cells, the functional roles and significance of miRNAs dysregulated in circulation in diabetes still need to be determined. Such studies could enable broader implementation of circulating miRNA biomarkers of β-cell dysfunction in combination with other relevant biomarker types (*e*.*g*., glucose/insulin levels, cytokine and/or autoantibody levels) for stratifying patients at an early stage – before clinical diabetes develops, predicting the progression of disease, guiding therapy, and/or monitoring responses to targeted interventions.

## Methods

### Study Design and Subject

For this cross-sectional study, we recruited subjects from the community, the Florida Hospital Diabetes Institute of Orlando, FL, and the University of Florida, Gainesville, FL. All studies and procedures were approved and carried out in accordance with the approved guidelines of the Florida Hospital Institutional Review Board and all subjects provided written informed consent prior to participation. Healthy control subjects (n = 27) had a body mass index (BMI) <30 kg/m^2^, had no history of diabetes, and were not on medications affecting glucose metabolism. Subjects with prediabetes (n = 12) had either impaired fasting glucose levels [fasting plasma glucose (FPG) in the range 100 mg/dL (5.6 mmol/L) to 125 mg/dL (6.9 mmol/L)], impaired glucose tolerance [2-h plasma glucose (2-h PG) value after a 75-g oral glucose tolerance test (OGTT) in the range 140 mg/dL (7.8 mmol/L) to 199 mg/dL (11.0 mmol/L)], or an HbA_1c_ of 5.7–6.4% (39–46 mmol/mol)^21^. Subjects with T2D (n = 31) had a prior diagnosis of T2D or a HbA_1c_ ≥6.5% (49 mmol/mol)^21^ and were treated with diet/exercise or monotherapy with metformin, sulfonylureas, or DPP-4 inhibitors. Individuals on insulin or other anti-hyperglycemic agents were excluded. LADA subjects (n = 6) were diagnosed after age 30 y, had a BMI <30 kg/m^2^, had positive glutamic acid decarboxylase (GAD65) antibodies, islet cell antibodies (ICA) or insulin antibodies (IAA) and had not been treated with insulin during the first 6 months after diagnosis. T1D subjects (n = 16) had a clinical diagnosis of T1D with onset before 30 years of age, had a BMI <30 kg/m^2^, were positive for GAD65, ICA or IAA antibodies and were treated with insulin from the time of diagnosis.

### Clinical and Metabolic Testing

All testing was performed at the Florida Hospital Translational Research Institute Clinical Research Unit (CRU). Anthropometric measures (weight, height, waist circumference) were performed with the subjects in a light hospital gown according to standardized protocols. Body composition was measured by Dual Energy X-Ray Absorptiometry using a GE Lunar iDXA whole-body scanner (Lunar iDEXA, GE, Madison, WI, USA). After fasting, blood samples were obtained, subjects underwent a 2-hour 75 g OGTT. Subjects with LADA and T1D took their basal insulin the evening before, but withheld prandial insulin the morning of the OGTT. Subjects with T2D withheld all medications the morning of testing. After the OGTT, subjects were fed lunch, received insulin coverage if necessary and were discharged from the CRU when their blood glucose had stabilized.

Plasma glucose concentrations were measured by the glucose oxidase method using the YSI 2300 STAT Plus Analyzer (YSI Life Sciences). Plasma insulin and C-peptide concentrations were determined using the MSD human insulin assay kit (K151BZC) and C-peptide kit (N45CA-1), respectively (Meso Scale Discovery, Inc.). HbA_1c_ levels were measured using the Cobas Integra 800 (Roche) immunoassay. β-cell function was assessed by calculating HOMA-B, the insulinogenic index [ΔI-30′/ΔG-30′] and the insulin and c-peptide areas under the curve in response to the OGTT. Insulin action was assessed by calculating HOMA-IR and the Matsuda, Quicki and ISSI-2 indices as described^22^,^23^. Insulin levels were not measured in subjects with LADA and T1D as they were treated with exogenous insulin.

### miRNA profiling

Fasting venous blood samples were collected in EDTA treated vacutainer tubes. Plasma (200 μL) was added to 1 mL of QIAzol Lysis buffer and then spiked with 3.5 μL of miRNeasy Serum/Plasma Spike-In Control (Cel-miR-39, 1.6 × 10^8^ copies/μL working solution). Total RNA was extracted using miRNeasy Serum/Plasma Kit (QIAGEN, 217184) following manufacturer’s instructions and treated with DNase I. A reverse transcription (RT) primer pool was created with specific miRNA RT primers and qRT-PCR was performed to measure the miRNAs. Briefly, 3 μL of RNA was added into each well containing 6 μL of the 1:100 diluted TaqMan^®^ MicroRNA Assays 5xRT primer pool and RT reaction mix for a total reaction volume of 15 μL using TaqMan^®^ MicroRNA RT kit (Life Technologies, 4366596) and incubated as indicated by the manufacturer’s protocol. The cDNA was then pre-amplified through 12 cycles using 3.75 μL of 1:100 diluted pool of primers (20× TaqMan^®^ MicroRNA Assays), 2.5 μL RT Product and 12.5 μL TaqMan^®^ PreAmp Master Mix (2X) in a final volume of 25 μL following manufacturer’s protocol (Life Tech., 4391128) and amplified per manufacturer’s instructions. For real-time PCR reactions, 0.1 μL of the 1:8 diluted preamplification products were amplified at a final volume of 10 μL by qRT-PCR with 5 μL of 2× TaqMan^®^ Universal Master Mix II (Life Tech., 4440048), 0.5 μL of 20 × TaqMan^®^ MicroRNA Assay following manufacturer’s instructions.

### Binary and Multi-class Classification using Random Forest

Random Forest (RF) classification (using the randomForest package), estimation of diagnostic odds ratio (DOR), and sensitivity analysis (using the ROCR package) were implemented in the R environment. The adjusted normalized expression level (adj.logFC) of differentially abundant circulating miRNAs was used to evaluate the biomarker potential of miRNAs in multiple combinations. RF generates importance measures (*e*.*g*., Gini scores) for the features used as predictor variables, which are helpful for feature selection. Based on the Gini scores of variable importance obtained from an initial RF run including all differentially abundant circulating miRNAs as predictor variables, we then recursively ran the RF algorithm with only a subset of the miRNA predictors, starting with the combination of the top 2, then top 3, top 4, and so forth, to generate seven distinct RF classifiers. We then selected the RF classifiers with the lower out-of-bag (OOB) estimate of error rate for further performance evaluation using the ROCR package. RF was implemented in two ways: (1) as a binary classification approach to validate the existence of miRNA signatures that can differentiate each disease subtype from healthy controls, and (2) as a multi-class classification approach to assess the practical diagnostic value of circulating miRNAs for simultaneous differentiation of all five study groups (that include 4 disease groups/subtypes and the Healthy group). The randomForest() function was used with default mtry and cutoff parameters [mtry is the number of predictor variables randomly sampled as candidates at each tree split with default value equal to sqrt(p), where p is number of predictor variables; cutoff is a vector of length equal to the number of classes and default value equal to 1/k, where k is the number of classes (the ‘winning’ class for an observation is the one with the maximum ratio of proportion of votes to cutoff)], ntree = 5001 (number of trees to grow), importance = TRUE (to assess importance of predictors), and proximity = TRUE [to calculate proximity among the rows (samples) and be able to generate multidimensional scaling (MDS) plots using the function MDSplot()]. The sample size parameter that defines the sizes of sample to draw for random tree generation was defined as c(6, 6) for binary classifications and c(6, 6, 6, 6, 6) for multi-class classifications [that is a vector of length equal to the respective number of classes, and value equal to the minimum number of observations per class, which is 6, the number of subjects in the LADA class). The RF prediction probabilities were generated using the function predict() on the RF object while setting the argument type = “prob”. To evaluate the performance of the RF classifiers, we used the ROCR package. For this, we first needed to apply the ROCR prediction() function on the matrix of RF prediction probabilities to create a prediction object that transform these probabilities into a standardized format matrix. Since ROCR supports only binary classifications, for generation of the multi-class ROCR prediction object, the vector of 5-class labels was transformed into a vector of 2-class labels (*e*.*g*., vector of T2D and All-Others labels) using the mapvalues() function from the plyr package. This is equivalent to say that the 5-class classification problem was reformulated into five separate all-*versus*-one (OVA) comparisons for the purpose of sensitivity analysis. The performance() function was then used to generate performance measures and curves. For ROC curve visualization, the true positive rate (sensitivity) was plotted as a function of the false positive rate (1-specificity). DORs were calculated for each OVA comparison using the formula DOR = (TP/FN)/(FP/TN), where TP is the number of true positives, FN, the number of false negatives, FP, the number of false positives, and TN, the number of true negatives. DOR of a test is a single indicator of diagnostic performance and represents the odds of positivity in individuals with the disease relative to the odds of positivity in individuals without the disease.

### Statistical Analyses

All data were entered into a custom research database (Cerner DiscoverE) and analyzed with Statistical Analysis System (SAS) version 9.3 (SAS Institute, Cary, NC, USA), Bioconductor packages (*e*.*g*., limma, randomForest, and ROCR) in the R environment, and GraphPad Prism version 6.0 (GraphPad Software Inc., La Jolla, CA, USA). The fold change for miRNA expression was obtained using the 2^−∆∆Ct^ method^24^,^25^. The geometric mean^26^ of two endogenous control miRNAs (hsa-miR-191 and miR-451) was used for sample-to-sample normalization. Differential abundance of miRNAs in plasma was assessed using Bayesian statistics implemented in the limma R package. To correct for confounding effects of age, gender, and BMI imbalances, these variables were included in the linear model used by limma. Clinical and biochemical data are presented as mean ± SEM. Differences between groups were analyzed by one-way ANOVA and *post-hoc* group comparisons performed with the Tukey-Kramer adjustment. Correlation analyses were performed by calculating Spearman and Pearson correlation coefficients and by calculating partial correlation coefficients adjusting for BMI, age, and gender. Statistical significance was considered at *p*-value < 0.05. Adjusted *p* values (FDR) were also calculated and reported for guidance while assessing potential impact of multiple testing.

## Additional Information

**How to cite this article**: Seyhan, A. A. *et al.* Pancreas-enriched miRNAs are altered in the circulation of subjects with diabetes: a pilot cross-sectional study. *Sci. Rep.* **6**, 31479; doi: 10.1038/srep31479 (2016).

## Supplementary Material

Supplementary Information

## Figures and Tables

**Table 1:** Demographic and clinical characteristics of study cohorts.

	Healthy (n = 27)	Prediabetes (n = 12)	T2D (n = 31)	LADA (n = 6)	T1D (n = 16)	*p*-value
Gender (M/F)	15/12	7/5	15/16	4/2	7/9	0.8060
Age (years)	25.3 ± 2.2^a,b,c^	40.8 ± 3.0^a,g^	52.9 ± 2.0^c,j^	54.0 ± 3.2^b,i^	25.9 ± 5.7^g,i,j^	<0.0001
BMI kg/m^2^	24.1 ± 0.9^a,c^	30.8 ± 3.1^a,g^	34.1 ± 1.3^c,h,j^	24.3 ± 1.2^h^	19.9 ± 1.2^g,j^	<0.0001
Height (cm)	167 ± 3^d^	172 ± 4^g^	169 ± 6^j^	170 ± 2^i^	146 ± 7^d,g,i,j^	0.0001
Weight (kg)	68.8 ± 3.6^a,c^	94.2 ± 12.0^a,g^	69.9±7.4^c,j^	99.1 ± 4.5	46.4 ± 6.0^g,j^	<0.0001
Hb-A1C (%)	5.24 ± 0.06^b,c,d^	5.75 ± 0.09^e,f,g^	6.56 ± 0.11^c,f,h,j^	8.85 ± 0.83^b,e,,h,i^	7.63 ± 0.39^d,g,i^	<0.0001
Glucose baseline (mg/dl)	87.5 ± 2.0^c^	96.2 ± 3.3^f^	184.9 ± 16.5^c,f^	126.1 ± 4.7^i^	124.2 ± 17.2^i^	<0.0001
Insulin baseline (μIU/ml)	5.48 ± 0.86^c^	7.74 ± 2.35	12.40 ± 2.10^c^	NA	NA	0.0345
OGTT-glucose, AUC (mg/dl × min)	14435 ± 508	19089 ± 908^f,g^	29439 ± 1613^f^	37411 ± 2137	33059 ± 1315^g^	<0.0001
OGTT-c-peptide, AUC (ng/ml × min)	678.6 ± 96.9^b,d^	921.4 ± 135.9^e,g^	801.1 ± 85.9^h^	60.4 ± 18.5^b,e,h^	39.0 ± 26.3^d,g^	<0.0001
OGTT-insulin, AUC (μIU/ml × min)	4525 ± 1239	8402 ± 2218	7189 ± 1190	NA	NA	0.3934
ΔIns.30 min/ΔGlu.30 min	0.88 ± 0.40	0.61 ± 0.23	0.42 ± 0.10	NA	NA	0.2831
ISSI2	3.03 ± 0.40^a,c^	1.83 ± 0.18^a,f^	0.88 ± 0.12^c,f^	NA	NA	<0.0001
HOMA-B	42.72 ± 10.06	81.06 ± 25.69	67.92 ± 16.89	NA	NA	0.5722
HOMA-IR	1.19 ± 0.19^c^	1.89 ± 0.56	4.08 ± 0.75^c^	NA	NA	0.0052
MATSUDA	15.56 ± 3.65^c^	10.61 ± 2.72	6.04 ± 1.30^c^	NA	NA	0.0167
QUICKI	0.44 ± 0.02^c^	0.41 ± 0.03	0.36 ± 0.02^c^	NA	NA	0.0258

Due to the confounding effect of exogenous insulin, LADA and T1D patients were not included in analyses of insulin secretion and sensitivity that relied on endogenous insulin responses. Different letters in superscripts following each *p* value indicate statistical significance after the Tukey adjustment for multiple comparisons: ^a^Healthy *vs*. Prediabetes, ^b^Healthy *vs*. LADA, ^c^Healthy *vs*. T2D, ^d^Healthy *vs*. T1D, ^e^Prediabetes *vs*. LADA, ^f^Prediabetes *vs*. T2D, ^g^Prediabetes *vs*. T1D, ^h^LADA *vs*. T2D, ^i^LADA *vs*. T1D, ^j^T2D *vs*. T1D. NA: Not applicable.

**Table 2:** Summary of the differential abundance analysis for pancreatic miRNAs in circulation.

	logFC	FC	P.Value	adj.P.Val		logFC	FC	P.Value	adj.P.Val
**PreT2D-Healthy**					**LADA-Healthy**					
miR-126	−0.95	0.52	**0**.**0243**	**0**.**1217**	miR-126	−0.03	0.98	0.9512	0.9868	
miR-146a	−0.94	0.52	**0**.**0207**	**0**.**1217**	miR-146a	0.27	1.21	0.6127	0.9868	
miR-148a	−0.96	0.51	0.0608	0.1521	miR-148a	0.01	1.01	0.9868	0.9868	
miR-21	−0.43	0.74	0.2240	0.3734	miR-21	0.08	1.05	0.8715	0.9868	
miR-24	−0.73	0.60	0.0823	0.1647	miR-24	0.43	1.35	0.4336	0.9868	
miR-29a	−1.08	0.47	0.0530	0.1521	miR-29a	−0.64	0.64	0.3861	0.9868	
miR-30d	−0.12	0.92	0.8110	0.8110	miR-30d	−0.27	0.83	0.6800	0.9868	
miR-34a	−0.22	0.86	0.7658	0.8110	miR-34a	1.37	2.59	0.1717	0.9868	
miR-375	−0.62	0.65	0.4004	0.5005	miR-375	−0.26	0.84	0.7910	0.9868	
miR-376a	−0.62	0.65	0.2767	0.3953	miR-376a	0.16	1.12	0.8324	0.9868	
**T2D-Healthy**					**T1D-Healthy**					
miR-126	0.79	1.73	0.0506	**0**.**0894**	miR-126	0.48	1.39	0.1794	0.2243	
miR-146a	0.34	1.26	0.3828	0.3828	miR-146a	0.53	1.44	0.1243	0.1776	
miR-148a	0.97	1.96	**0**.**0481**	**0**.**0894**	miR-148a	1.11	2.15	**0**.**0119**	**0**.**0396**	
miR-21	0.74	1.67	**0**.**0301**	**0**.**0894**	miR-21	0.86	1.81	**0**.**0052**	**0**.**0396**	
miR-24	0.59	1.50	0.1428	0.2040	miR-24	0.85	1.81	**0**.**0181**	**0**.**0452**	
miR-29a	1.03	2.04	0.0537	**0**.**0894**	miR-29a	0.80	1.74	0.0920	0.1582	
miR-30d	1.41	2.66	**0**.**0034**	**0**.**0338**	miR-30d	0.70	1.63	0.0949	0.1582	
miR-34a	1.72	3.30	**0**.**0184**	**0**.**0894**	miR-34a	0.70	1.63	0.2723	0.3026	
miR-375	0.82	1.77	0.2460	0.3023	miR-375	1.68	3.21	**0**.**0085**	**0**.**0396**	
miR-376a	−0.60	0.66	0.2721	0.3023	miR-376a	0.36	1.28	0.4602	0.4602	

The differential abundance analysis was implemented in the R environment using the limma package. Features (miRNAs) are sorted alphabetically. Significant values [*p *< 0.05 and/or adjusted *p* value (FDR) < 0.13] are highlighted in bold and underlined.
