# Supplementary material for: Pancreas-enriched miRNAs are altered in the circulation of subjects with diabetes: a pilot cross-sectional study
Source: Sci Rep. 2016 Aug 25;6:31479. doi: 10.1038/srep31479 (PMC4997329; doi:10.1038/srep31479)
Supplement: Supplementary Information [file srep31479-s1.doc]

# Pancreas-enriched miRNAs are altered in the circulation of subjects with diabetes: a pilot cross sectional study

Attila Seyhan^1, 2, 3^, *, Yury O. Nunez Lopez^1^, Hui Xie^1^, Fanchao Yi^1^, Clayton Mathews^4^, Magdalena Pasarica^5^ and Richard E. Pratley^1, 3^

**Supplementary Figure 1. Day to day reproducibility of detection and effects of glucose on the stability of circulating miRNAs.**


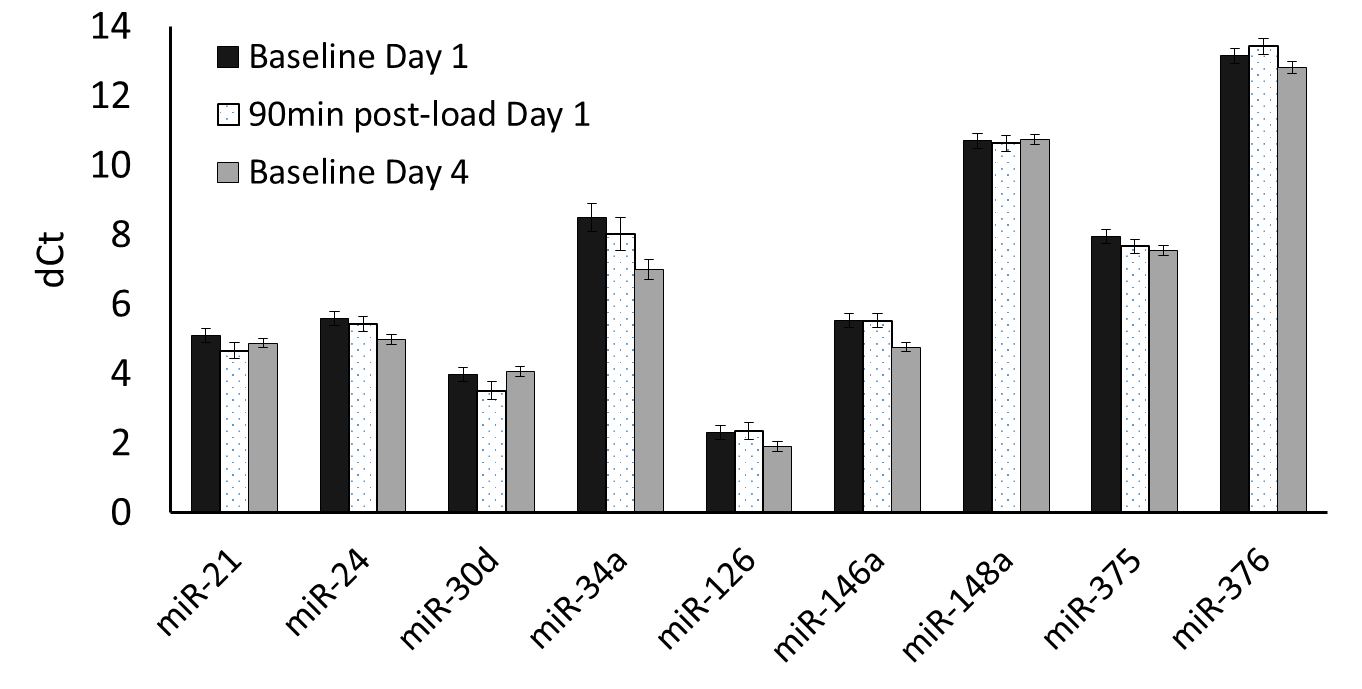


**Supplementary Figure 1. Day to day reproducibility of detection and effects of glucose on the stability of circulating miRNAs.** To test the reproducibility of circulating miRNA expression levels, selected miRNAs were measured in fasting samples collected on two days separated by 3 days in a subset of subjects with T2D (n =12). We also tested the effects of acute increases in circulating glucose levels by measuring miRNA expression in a subset of fasting samples and samples collected at the 90 min (n =12) time point during the OGTT. For these analyses, all samples from individual subjects were measured in a single assay. These analyses confirmed that the expression of miRNAs was stable from day to day and was not significantly (P>0.05) affected by acute increases in glucose. The results were highly reproducible (CV: 4-13%).

**Statement of justification:** We think it is important to present our data showing the effects of acute increase in glucose on the stability of circulating miRNAs. Our findings show that the miRNAs were stable in fasting plasma samples collected on two different days (CV: 4%) and they did not change with acute changes in glucose during the OGTT (CV: 5%) in T2D subjects.

**Supplementary Table 1. miRNAs used in the study.** A panel of 28 miRNAs associated with β-cell function and diabetes were identified from the literature ^1-20^.

| **miRNA** | **Disease** | **miRNA assay** |
| --- | --- | --- |
| **miR-126** | Diabetes | hsa-miR-126 002228 |
| **miR138** | Diabetes | hsa-miR-138 002284 |
| **miR-146a** | Diabetes | hsa-miR-146a 000468 |
| **miR-150** | Diabetes | hsa-miR-150 000473 |
| **miR-15a** | Diabetes | hsa-miR-15a 000389 |
| **miR-223** | Diabetes | hsa-miR-223 002295 |
| **miR-27a** | Diabetes | hsa-miR-27a 000408 |
| **miR-28-3p** | Diabetes | hsa-miR-28-3p 002446 |
| **miR-29a** | Diabetes | hsa-miR-29b 002112 |
| **miR-29b** | Diabetes | hsa-miR-29b 000413 |
| **miR-30d** | Diabetes | hsa-miR-30d 000420 |
| **miR-31** | Diabetes | hsa-miR-31 002279 |
| **miR-320a** | Diabetes | hsa-miR-320 002277 |
| **miR-34a** | Diabetes | hsa-miR-34a 000426 |
| **miR-375** | Diabetes | hsa-miR-375 000564 |
| **miR-376a** | Diabetes | hsa-miR-376a 000565 |
| **miR-508** | Diabetes | hsa-miR-508 001052 |
| **miR-9** | Diabetes | hsa-miR-9 000583 |
| **miR‑148a** | Diabetes | hsa-miR-148a 000470 |
| **miR‑152** | Diabetes | hsa-miR-152 000475 |
| **miR‑155** | Diabetes | hsa-miR-155 002623 |
| **miR‑181a** | Diabetes | hsa-miR-181a 000480 |
| **miR‑199a** | Diabetes | hsa-miR-199a 000498 |
| **miR-21** | Diabetes | hsa-miR-21 000397 |
| **miR‑24.1** | Diabetes | hsa-miR-24.1 000402 |
| **miR‑25** | Diabetes | hsa-miR-25 000403 |
| **miR-326** | Diabetes | hsa-miR-326 000542 |
| **miR-93** | Diabetes | hsa-miR-93* 002139 |
| **miR-451** | Endogenous control | hsa-miR-451 |
| **miR-191** | Endogenous control | hsa-miR-191 002299 |
| **miR-423-3p** | Endogenous control | hsa-miR-423-3P 002626 |
| **RNU6** | Endogenous control | RNU6 001006 |
| **cel-miR-39** | Spike in control | cel-miR-39 000200 |

**Statement of justification:** We think it is important to present our initial panel of diabetes associated 28 miRNAs to the scientific community. This panel was created by extensive literature review involving large clinical studies. Our study showed that of the 28 miRNAs in the testing panel, 8 were significantly (P<0.05) altered in the plasma of different types of diabetes subjects and were present or enriched (*e.g.* miR-126, miR146a, miR-148a, miR-21, miR-24, miR-30d, miR-34a) or specific (*e.g.* miR-375) to pancreatic islet β-cells. From this, we have built a pancreas-enriched panel of miRNAs as “circulating potential biomarkers of β-cell injury” to both segment and assess disease progression, and potentially gauge therapeutic interventions that prevent, reduce, or delay disease progression.

**Supplementary Table 2. Summary of the functional roles of miRNAs that were elevated in the circulation in diabetes.**

| **miRNAs** | **Target tissue / organ** | **Function** | **Confirmed targets** | **References** |
| --- | --- | --- | --- | --- |
| **miR-375** | Pancreas | Insulin secretion and apoptosis by lipoapoptosis | PDK1, Mtpn, Vti1a  MTPN, USP1, JAK2, ADIPOR2 | 1-5 |
| **miR-21** | Pancreas, heart and vascular tissue | Smooth muscle survival and proliferation | ERK, PTEN, RhoB, PPAR, Bcl2, PDCD4 | 6-8 |
| **miR-30d** | Pancreas, beta-cell | Insulin biosynthesis |  | 9, 10 |
| **miR-34a** | Pancreas | Beta-cell apoptosis | VAMP2, Bcl2 | 11 |
| **miR-146a** | Pancreas | Beta-cell apoptosis |  | 11 |
| **miR-24.1** | Pancreas | Insulin synthesis | Sox6, Bhlhe22 | 12 |
| **miR-126** | Pancreas, skeletal muscle | Pancreatic development | SPRED1, PI3KR2 | 5, 13 |
| **miR-148a** | Pancreas | Insulin synthesis | Sox6, Bhlhe22 | 12 |

**Statement of justification:** Our data shows that miRNAs elevated in circulation in different types of diabetes play important roles ranging from glucose and insulin homeostasis to vascular integrity. Previous studies have identified and validated the targets that are regulated by these miRNAs emphasizing the need for linking these miRNAs to their biologic functions and their involvement in beta cell function, glucose and insulin homeostasis and endothelial functions.

**Supplementary Table 3**. Binary Random Forest classification (Healthy-versus-PreT2D) using 4 differentially abundant miRNAs (miR-146a, miR-126, miR-30d, miR-148a).

**Statement of justification:** We believe that presenting data from the Binary Random Forest classification will improve distinguishing the subtypes of diabetes.

**Supplementary Table 4**. Binary Random Forest classification (Healthy-versus-LADA) using 3 differentially abundant miRNAs (miR-34a, miR-24, miR-21)

**Statement of justification:** We believe that presenting data from the Binary Random Forest classification will improve distinguishing the subtypes of diabetes.

**Supplementary Table 5**. Binary Random Forest classification (Healthy-versus-T2D) using 2 differentially abundant miRNA predictors (miR-30d, miR-34a)

**Statement of justification:** We believe that presenting data from the Binary Random Forest classification will improve distinguishing the subtypes of diabetes.

**Supplementary Table 6**. Binary Random Forest classification (Healthy-versus-T1D) using 2 differentially abundant miRNA predictors (miR-21, miR-375)

**Statement of justification:** We believe that presenting data from the Binary Random Forest classification will improve distinguishing the subtypes of diabetes.

**Supplementary Table 7**. Multi-class Random Forest classification using 6 differentially abundant miRNAs (miR-30d, miR-21, miR-148a, miR-375, miR-126, miR-24)

**Statement of justification:** We believe that presenting data from the Binary Random Forest classification will improve distinguishing the subtypes of diabetes.

**Supplementary Table 8**. Multimodal multi-class Random Forest classification using fasting glucose levels and 2 differentially abundant miRNAs (Glucose, miR-30d, miR-21)

**Statement of justification:** We believe that presenting data from the Binary Random Forest classification will improve distinguishing the subtypes of diabetes.

**Supplementary** **Table 9. Partial correlation coefficients of circulating miRNA levels with glycemic control and clinical parameters in subjects from the Prediabetes group.** The first value in each column represents the partial correlation coefficient (r), the second value the significance (*p*), and the third number the sample size (n). Significant values (*p < 0.05*) are in bold.

| **PreT2D** | **miR-126** | **miR-146a** | **miR-148a** | **miR-21** | **miR-24** | **miR-29a** | **miR-30d** | **miR-34a** | **miR-375** | **miR-376a** |  |
| --- | --- | --- | --- | --- | --- | --- | --- | --- | --- | --- | --- |
| **AUC-Glucose** | 0.49 | 0.45 | 0.6 | 0.45 | 0.57 | 0.37 | 0.24 | 0.55 | 0.14 | 0.13 | *r* |
|  | 0.1076 | 0.144 | **0.0377** | 0.1436 | 0.0537 | 0.2365 | 0.4435 | 0.0666 | 0.6726 | 0.6912 | *p* |
|  | 12 | 12 | 12 | 12 | 12 | 12 | 12 | 12 | 12 | 12 | *n* |
| **AUC-Insulin** | 0.48 | 0.46 | 0.4 | 0.51 | 0.43 | 0.57 | 0.42 | 0.76 | 0.62 | 0.46 | *r* |
|  | 0.1213 | 0.1338 | 0.1996 | 0.0936 | 0.1689 | 0.0555 | 0.1774 | **0.0059** | **0.0326** | 0.1338 | *p* |
|  | 12 | 12 | 12 | 12 | 12 | 12 | 12 | 12 | 12 | 12 | *n* |
| **AUC-c-peptide** | 0.39 | 0.4 | 0.55 | 0.43 | 0.39 | 0.56 | 0.58 | 0.64 | 0.6 | 0.53 | *r* |
|  | 0.2042 | 0.201 | 0.0707 | 0.1631 | 0.2126 | 0.0574 | **0.0466** | **0.0263** | **0.0392** | 0.0793 | *p* |
|  | 12 | 12 | 12 | 12 | 12 | 12 | 12 | 12 | 12 | 12 | *n* |
| **HbA1c** | 0.69 | 0.54 | 0.23 | 0.64 | 0.49 | 0.55 | 0.29 | 0.59 | 0.18 | -0.15 | *r* |
|  | **0.0134** | 0.0703 | 0.4698 | **0.0244** | 0.102 | 0.0655 | 0.3584 | **0.0421** | 0.5854 | 0.6397 | *p* |
|  | 12 | 12 | 12 | 12 | 12 | 12 | 12 | 12 | 12 | 12 | *n* |
| **HOMA-B** | 0.41 | 0.33 | 0.32 | 0.44 | 0.3 | 0.55 | 0.59 | 0.69 | 0.64 | 0.33 | *r* |
|  | 0.1926 | 0.2974 | 0.3181 | 0.1542 | 0.3425 | 0.0666 | **0.0422** | **0.0159** | **0.028** | 0.2974 | *p* |
|  | 12 | 12 | 12 | 12 | 12 | 12 | 12 | 12 | 12 | 12 | *n* |
| **HOMA-IR** | 0.54 | 0.39 | 0.46 | 0.57 | 0.42 | 0.61 | 0.59 | 0.87 | 0.61 | 0.15 | *r* |
|  | 0.0749 | 0.2096 | 0.1354 | 0.0555 | 0.1766 | **0.04** | **0.0419** | **0.0004** | **0.0366** | 0.6351 | *p* |
|  | 12 | 12 | 12 | 12 | 12 | 12 | 12 | 12 | 12 | 12 | *n* |
| **MATSUDA** | -0.63 | -0.52 | -0.7 | -0.65 | -0.63 | -0.67 | -0.54 | -0.89 | -0.56 | -0.36 | *r* |
|  | **0.0274** | 0.0834 | **0.0112** | **0.0212** | **0.0289** | **0.0168** | 0.0678 | **0.0001** | 0.0627 | 0.256 | *p* |
|  | 12 | 12 | 12 | 12 | 12 | 12 | 12 | 12 | 12 | 12 | *n* |
| **QUICKI** | -0.62 | -0.49 | -0.72 | -0.65 | -0.58 | -0.68 | -0.57 | -0.87 | -0.55 | -0.22 | *r* |
|  | **0.0328** | 0.1043 | **0.0088** | **0.0214** | **0.0465** | **0.0155** | 0.0539 | **0.0004** | 0.0666 | 0.4938 | *p* |
|  | 12 | 12 | 12 | 12 | 12 | 12 | 12 | 12 | 12 | 12 | *n* |
| **ISSI2** | -0.58 | -0.48 | -0.76 | -0.62 | -0.65 | -0.48 | -0.46 | -0.67 | -0.14 | 0.12 | *r* |
|  | 0.0521 | 0.1154 | **0.0041** | **0.0348** | **0.0259** | 0.1154 | 0.1339 | **0.0204** | 0.6672 | 0.7118 | *p* |
|  | 12 | 12 | 12 | 12 | 12 | 12 | 12 | 12 | 12 | 12 | *n* |
| **Ins30-glu30** | 0.47 | 0.36 | 0.42 | 0.52 | 0.29 | 0.66 | 0.7 | 0.44 | 0.62 | 0.56 | *r* |
|  | 0.1275 | 0.2463 | 0.1766 | 0.0887 | 0.3543 | **0.024** | **0.0118** | 0.1542 | **0.0373** | 0.0627 | *p* |
|  | 12 | 12 | 12 | 12 | 12 | 12 | 12 | 12 | 12 | 12 | *n* |

**Supplementary** **Table 10. Partial correlation coefficients of circulating miRNA levels with glycemic control and clinical parameters in subjects from the Healthy group.** The first value in each column represents the partial correlation coefficient (r), the second value the significance (*p*), and the third number the sample size (n). Significant values (*p < 0.05*) are in bold.

| **HEALTHY** | **miR-126** | **miR-146a** | **miR-148a** | **miR-21** | **miR-24** | **miR-29a** | **miR-30d** | **miR-34a** | **miR-375** | **miR-376a** |  |
| --- | --- | --- | --- | --- | --- | --- | --- | --- | --- | --- | --- |
| **AUC-Glucose** | -0.62 | -0.55 | -0.7 | -0.55 | -0.54 | -0.59 | -0.09 | -0.43 | -0.75 | 0.04 | *r* |
|  | **0.0321** | 0.0666 | **0.0145** | 0.0666 | 0.0749 | **0.0488** | 0.7832 | 0.1614 | **0.0047** | 0.9037 | *p* |
|  | 12 | 12 | 12 | 12 | 12 | 12 | 12 | 12 | 12 | 12 | *n* |
| **AUC-Insulin** | 0.29 | 0.49 | -0.13 | 0.38 | 0.27 | 0.13 | -0.47 | 0.28 | 0.26 | 0.33 | *r* |
|  | 0.3659 | 0.1074 | 0.6834 | 0.2275 | 0.4008 | 0.679 | 0.1275 | 0.3776 | 0.4098 | 0.2974 | *p* |
|  | 12 | 12 | 12 | 12 | 12 | 12 | 12 | 12 | 12 | 12 | *n* |
| **AUC-c-peptide** | 0.22 | 0.46 | 0.13 | 0.39 | 0.34 | 0.27 | 0.19 | 0.56 | -0.3 | -0.52 | *r* |
|  | 0.4849 | 0.1332 | 0.6997 | 0.2096 | 0.2867 | 0.404 | 0.5578 | 0.0627 | 0.3435 | 0.0887 | *p* |
|  | 12 | 12 | 12 | 12 | 12 | 12 | 12 | 12 | 12 | 12 | *n* |
| **HbA1c** | -0.03 | -0.16 | 0.35 | 0.2 | -0.06 | 0.29 | 0.57 | 0.15 | -0.34 | -0.21 | *r* |
|  | 0.9327 | 0.6237 | 0.267 | 0.5382 | 0.8592 | 0.3527 | 0.0543 | 0.6423 | 0.2807 | 0.5085 | *p* |
|  | 12 | 12 | 12 | 12 | 12 | 12 | 12 | 12 | 12 | 12 | *n* |
| **HOMA-B** | 0.61 | 0.55 | 0.51 | 0.56 | 0.47 | 0.56 | 0.21 | -0.18 | -0.09 | 0.47 | *r* |
|  | **0.0024** | **0.008** | **0.0138** | **0.0061** | **0.0247** | **0.0064** | 0.3312 | 0.4072 | 0.6791 | **0.0251** | *p* |
|  | 23 | 23 | 23 | 23 | 23 | 23 | 23 | 23 | 23 | 23 | *n* |
| **HOMA-IR** | 0.59 | 0.53 | 0.53 | 0.54 | 0.44 | 0.53 | 0.18 | -0.32 | -0.11 | 0.59 | *r* |
|  | **0.0037** | **0.0103** | **0.0098** | **0.0091** | **0.0352** | **0.0108** | 0.4 | 0.1409 | 0.6149 | **0.0036** | *p* |
|  | 23 | 23 | 23 | 23 | 23 | 23 | 23 | 23 | 23 | 23 | *n* |
| **MATSUDA** | -0.09 | -0.28 | 0.08 | -0.2 | -0.29 | -0.03 | 0.5 | -0.09 | -0.22 | -0.31 | *r* |
|  | 0.7907 | 0.3868 | 0.8173 | 0.5291 | 0.3543 | 0.9212 | 0.0988 | 0.7853 | 0.4849 | 0.3195 | *p* |
|  | 12 | 12 | 12 | 12 | 12 | 12 | 12 | 12 | 12 | 12 | *n* |
| **QUICKI** | -0.59 | -0.53 | -0.53 | -0.54 | -0.44 | -0.53 | -0.14 | 0.31 | 0.08 | -0.59 | *r* |
|  | **0.0037** | **0.0103** | **0.0098** | **0.0091** | **0.0352** | **0.0108** | 0.5334 | 0.1558 | 0.7092 | **0.0036** | *p* |
|  | 23 | 23 | 23 | 23 | 23 | 23 | 23 | 23 | 23 | 23 | *n* |
| **ISSI2** | 0.38 | 0.46 | 0.2 | 0.31 | 0.32 | 0.28 | 0.01 | 0.46 | 0.3 | -0.18 | *r* |
|  | 0.217 | 0.1318 | 0.5284 | 0.3209 | 0.3178 | 0.3741 | 0.9737 | 0.1338 | 0.338 | 0.5758 | *p* |
|  | 12 | 12 | 12 | 12 | 12 | 12 | 12 | 12 | 12 | 12 | *n* |
| **Ins30-glu30** | 0.4 | 0.46 | 0.29 | 0.5 | 0.44 | 0.4 | -0.1 | 0.37 | 0.31 | -0.17 | *r* |
|  | 0.1981 | 0.1282 | 0.3543 | 0.0993 | 0.1542 | 0.201 | 0.7495 | 0.2358 | 0.3244 | 0.5918 | *p* |
|  | 12 | 12 | 12 | 12 | 12 | 12 | 12 | 12 | 12 | 12 | *n* |

**Supplementary** **Table 11. Partial correlations of circulating miRNA levels with glycemic control and clinical parameters in subjects from the Diabetes group.** The first value in each column represents the partial correlation coefficient (r), the second value the significance (*p*), and the third number the sample size (n). Significant values (*p < 0.05*) are in bold.

| **T2D20** | **miR-126** | **miR-146a** | **miR-148a** | **miR-21** | **miR-24** | **miR-29a** | **miR-30d** | **miR-34a** | **miR-375** | **miR-376a** |  |
| --- | --- | --- | --- | --- | --- | --- | --- | --- | --- | --- | --- |
| **AUC-Glucose** | 0.44 | 0.33 | 0.41 | 0.46 | 0.37 | 0.38 | 0.45 | 0.16 | 0.51 | 0.24 | *r* |
|  | **0.0395** | 0.1353 | 0.0569 | **0.0297** | 0.0896 | 0.0818 | **0.0339** | 0.4821 | **0.0143** | 0.2761 | *p* |
|  | 22 | 22 | 22 | 22 | 22 | 22 | 22 | 22 | 22 | 22 | *n* |
| **AUC-Insulin** | 0.2 | -0.11 | 0.22 | 0.27 | -0.17 | 0.19 | 0.47 | 0.23 | 0.31 | 0.03 | *r* |
|  | 0.3585 | 0.6382 | 0.3245 | 0.2206 | 0.4502 | 0.3833 | **0.0277** | 0.2925 | 0.1539 | 0.8799 | *p* |
|  | 22 | 22 | 22 | 22 | 22 | 22 | 22 | 22 | 22 | 22 | *n* |
| **AUC-c-peptide** | -0.39 | -0.44 | -0.07 | -0.26 | -0.59 | -0.2 | 0.33 | 0.05 | 0.07 | -0.34 | *r* |
|  | 0.2104 | 0.1546 | 0.8211 | 0.4073 | **0.045** | 0.5412 | 0.3003 | 0.8802 | 0.8287 | 0.2804 | *p* |
|  | 12 | 12 | 12 | 12 | 12 | 12 | 12 | 12 | 12 | 12 | *n* |
| **HbA1c** | 0.04 | 0.15 | 0.08 | -0.01 | 0.22 | 0.04 | -0.2 | 0.31 | 0.1 | -0.19 | *r* |
|  | 0.8551 | 0.5181 | 0.7353 | 0.9761 | 0.3197 | 0.8463 | 0.3685 | 0.157 | 0.6646 | 0.3965 | *p* |
|  | 22 | 22 | 22 | 22 | 22 | 22 | 22 | 22 | 22 | 22 | *n* |
| **HOMA-B** | -0.08 | -0.2 | 0.16 | -0.16 | -0.27 | 0.01 | 0.27 | -0.52 | 0.09 | 0.31 | *r* |
|  | 0.6931 | 0.3136 | 0.4304 | 0.4292 | 0.171 | 0.9594 | 0.1729 | **0.0058** | 0.6554 | 0.1184 | *p* |
|  | 27 | 27 | 27 | 27 | 27 | 27 | 27 | 27 | 27 | 27 | *n* |
| **HOMA-IR** | -0.11 | -0.2 | -0.19 | -0.2 | -0.26 | -0.15 | 0.11 | -0.32 | -0.17 | 0.22 | *r* |
|  | 0.5893 | 0.3151 | 0.3335 | 0.3179 | 0.1906 | 0.4564 | 0.586 | 0.1066 | 0.4072 | 0.2608 | *p* |
|  | 27 | 27 | 27 | 27 | 27 | 27 | 27 | 27 | 27 | 27 | *n* |
| **MATSUDA** | 0.45 | 0.59 | -0.13 | 0.27 | 0.62 | 0.31 | -0.54 | -0.39 | -0.29 | 0.46 | *r* |
|  | 0.1472 | **0.0458** | 0.6892 | 0.3912 | **0.0373** | 0.3309 | 0.0695 | 0.2054 | 0.3621 | 0.1293 | *p* |
|  | 12 | 12 | 12 | 12 | 12 | 12 | 12 | 12 | 12 | 12 | *n* |
| **QUICKI** | 0.1 | 0.2 | 0.19 | 0.19 | 0.27 | 0.1 | -0.11 | 0.22 | 0.1 | -0.13 | *r* |
|  | 0.6115 | 0.3151 | 0.3335 | 0.3431 | 0.1661 | 0.6212 | 0.586 | 0.2654 | 0.6288 | 0.5319 | *p* |
|  | 27 | 27 | 27 | 27 | 27 | 27 | 27 | 27 | 27 | 27 | *n* |
| **ISSI2** | 0.13 | 0.06 | 0.1 | -0.17 | 0.22 | 0.22 | 0.22 | -0.2 | -0.15 | 0.05 | *r* |
|  | 0.6834 | 0.8517 | 0.7663 | 0.6039 | 0.4991 | 0.4849 | 0.4991 | 0.5292 | 0.6431 | 0.8679 | *p* |
|  | 12 | 12 | 12 | 12 | 12 | 12 | 12 | 12 | 12 | 12 | *n* |
| **Ins30-glu30** | -0.51 | -0.59 | -0.15 | -0.44 | -0.52 | -0.29 | 0.55 | 0.24 | -0.39 | -0.5 | *r* |
|  | 0.0889 | **0.0421** | 0.6498 | 0.1509 | 0.0813 | 0.3664 | 0.0666 | 0.4571 | 0.216 | 0.1041 | *p* |
|  | 12 | 12 | 12 | 12 | 12 | 12 | 12 | 12 | 12 | 12 | *n* |

**References**

1. Erener, S., Mojibian, M., Fox, J.K., Denroche, H.C. & Kieffer, T.J. Circulating miR-375 as a biomarker of beta-cell death and diabetes in mice. *Endocrinology* **154**, 603-8 (2013).

2. Poy, M.N. *et al.* A pancreatic islet-specific microRNA regulates insulin secretion. *Nature* **432**, 226-30 (2004).

3. El Ouaamari, A. *et al.* miR-375 targets 3'-phosphoinositide-dependent protein kinase-1 and regulates glucose-induced biological responses in pancreatic beta-cells. *Diabetes* **57**, 2708-17 (2008).

4. Heneghan, H.M., Miller, N. & Kerin, M.J. Role of microRNAs in obesity and the metabolic syndrome. *Obes Rev* **11**, 354-61 (2010).

5. Fernandez-Valverde, S.L., Taft, R.J. & Mattick, J.S. MicroRNAs in beta-cell biology, insulin resistance, diabetes and its complications. *Diabetes* **60**, 1825-31 (2011).

6. Creemers, E.E., Tijsen, A.J. & Pinto, Y.M. Circulating microRNAs: novel biomarkers and extracellular communicators in cardiovascular disease? *Circ Res* **110**, 483-95 (2012).

7. Etheridge, A., Lee, I., Hood, L., Galas, D. & Wang, K. Extracellular microRNA: a new source of biomarkers. *Mutat Res* **717**, 85-90 (2011).

8. Sabatel, C. *et al.* MicroRNA-21 exhibits antiangiogenic function by targeting RhoB expression in endothelial cells. *PLoS One* **6**, e16979 (2011).

9. Guay, C., Roggli, E., Nesca, V., Jacovetti, C. & Regazzi, R. Diabetes mellitus, a microRNA-related disease? *Transl Res* **157**, 253-64 (2011).

10. Shantikumar, S., Caporali, A. & Emanueli, C. Role of microRNAs in diabetes and its cardiovascular complications. *Cardiovasc Res* **93**, 583-93 (2012).

11. Lovis, P., Gattesco, S. & Regazzi, R. Regulation of the expression of components of the exocytotic machinery of insulin-secreting cells by microRNAs. *Biol Chem* **389**, 305-12 (2008).

12. Melkman-Zehavi, T. *et al.* miRNAs control insulin content in pancreatic beta-cells via downregulation of transcriptional repressors. *EMBO J* **30**, 835-45 (2011).

13. Zampetaki, A. & Mayr, M. MicroRNAs in vascular and metabolic disease. *Circ Res* **110**, 508-22 (2012).

14. Karolina, D.S. *et al.* Circulating miRNA profiles in patients with metabolic syndrome. *J Clin Endocrinol Metab* **97**, E2271-6 (2012).

15. Nielsen, L.B. *et al.* Circulating levels of microRNA from children with newly diagnosed type 1 diabetes and healthy controls: evidence that miR-25 associates to residual beta-cell function and glycaemic control during disease progression. *Exp Diabetes Res* **2012**, 896362 (2012).

16. Pescador, N. *et al.* Serum circulating microRNA profiling for identification of potential type 2 diabetes and obesity biomarkers. *PLoS One* **8**, e77251 (2013).

17. Salas-Perez, F. *et al.* MicroRNAs miR-21a and miR-93 are down regulated in peripheral blood mononuclear cells (PBMCs) from patients with type 1 diabetes. *Immunobiology* **218**, 733-7 (2013).

18. Sebastiani, G. *et al.* MicroRNA expression fingerprint in serum of type 1 diabetic patients. *Diabetologia* **55**, S48 (2012).

19. Kong, L. *et al.* Significance of serum microRNAs in pre-diabetes and newly diagnosed type 2 diabetes: a clinical study. *Acta Diabetol* **48**, 61-9 (2011).

20. Zampetaki, A. *et al.* Plasma microRNA profiling reveals loss of endothelial miR-126 and other microRNAs in type 2 diabetes. *Circ Res* **107**, 810-7 (2010)
